# Antioxidant Activity and the Therapeutic Effect of Sinomenine Hydrochloride-Loaded Liposomes-in-Hydrogel on Atopic Dermatitis

**DOI:** 10.3390/ijms25147676

**Published:** 2024-07-12

**Authors:** Xue Chen, Yang Wu, Ruoyang Jia, Yuqing Fang, Keang Cao, Xinying Yang, Xiaobo Qu, Hongmei Xia

**Affiliations:** College of Pharmacy, Anhui University of Chinese Medicine, Hefei 230012, China; 2023205221004@stu.ahtcm.edu.cn (X.C.); 2023205227034@stu.ahtcm.edu.cn (Y.W.); 2022205221003@stu.ahtcm.edu.cn (R.J.); 2023205221005@stu.ahtcm.edu.cn (Y.F.); 2023205221003@stu.ahtcm.edu.cn (K.C.); 2020205227037@stu.ahtcm.edu.cn (X.Y.); xiaoboqu2022@163.com (X.Q.)

**Keywords:** sinomenine, liposomes-in-hydrogel, colloidal hydrogel, anti-inflammatory, atopic dermatitis

## Abstract

Sinomenine hydrochloride is an excellent drug with anti-inflammatory, antioxidant, immune-regulatory, and other functions. Atopic dermatitis is an inherited allergic inflammation that causes itchiness, redness, and swelling in the affected area, which can have a significant impact on the life of the patient. There are many therapeutic methods for atopic dermatitis, and sinomenine with immunomodulatory activity might be effective in the treatment of atopic dermatitis. In this study, the atopic dermatitis model was established in experimental mice, and physical experiments were carried out on the mice. In the experiment, sinomenine hydrochloride liposomes-in-hydrogel as a new preparation was selected for delivery. In this case, liposomes were dispersed in the colloidal hydrogel on a mesoscopic scale and could provide specific transfer properties. The results showed that the sinomenine hydrochloride-loaded liposomes-in-hydrogel system could effectively inhibit atopic dermatitis.

## 1. Introduction

Atopic dermatitis (AD) is a chronic, hereditary, recurrent, inflammatory skin disease that is clinically manifested by rashes, erythema, exudates, epidermal peeling, edema, and papules on the skin [1]. The disease occurs at all stages of life, with a higher prevalence among women than men in adulthood [2]. Modern medical research has shown that when various external factors (including exposure to hot and cold stimuli, chemicals, pollen and dust, infection) interact with various internal factors of the immune system (including family genetics, mental state, skin dysfunction), it triggers an abnormal response of the immune system, which ultimately leads to the development of AD [3,4]. The pathogenesis of AD is often accompanied by inflammation and oxidative stress responses, which produce a large number of free radicals, further aggravating skin damage [5]. The course of AD is divided into an acute phase and a chronic phase. The acute phase is the immune response mainly by T helper 2 cells (Th2), which manifests as Th2 cell infiltration, and the levels of interleukin (IL)-4, IL-5, and IL-13 were increased. The chronic phase is an immune response dominated by Th1 cells, manifested by delayed hypersensitivity through interferon-γ (IFN-γ) and IL-2 responses and excessive collagen accumulation, inducing skin thickening and tissue remodeling [6,7]. There is no good cure for this disease in Western medicine. Glucocorticoids and immunosuppressants are used to treat AD, which can temporarily alleviate the related symptoms, but the long-term effect is not ideal, and there are more adverse reactions [8,9]. Therefore, further development of drugs from traditional Chinese medicine is needed to effectively treat AD.

**Figure 1:**
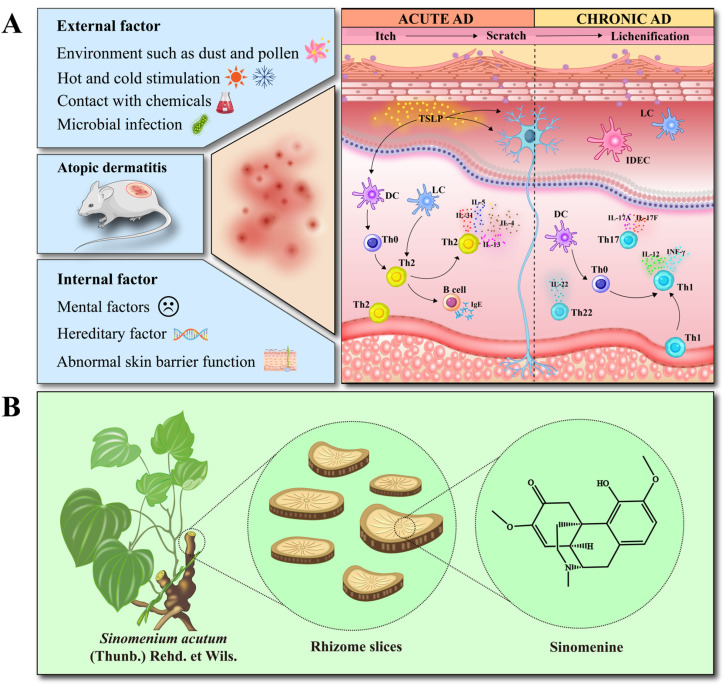
Pathogenesis of AD and source of SIN: (**A**) The sketch of the pathogenesis of atopic dermatitis, (**B**) Sinomenium acutum, rhizome slices, and the structure of sinomenine.

Sinomenine (SIN, C_19_H_23_NO_4_) (Figure 1) is the main component of the Chinese herb Sinomenium acutum (Thunb.) Rehder & E.H. Wilson. Because of the poor water solubility of SIN, the pharmaceutical industry mostly uses its sinomenine hydrochloride (SINH) as the water-soluble salt form of SIN. Experiments have proved that SINH has many biological activities such as anti-oxidation, anti-inflammation, immunomodulation, and improving microcirculation [10,11,12]. The existing clinical formulations of SINH are mainly used for rheumatoid arthritis and cardiac arrhythmias [13,14]. The mechanisms of action of SINH include regulating the balance of Th17/Treg cells, increasing the level of IL-10 (mainly secreted by Treg cells), and decreasing the level of IL-17A mainly secreted by Th17 cells [15]; decreasing the activity of antigen-presenting cells and affecting the nuclear factor κB (NF-κB) pathway [16]; inhibiting the production of serum pro-inflammatory cytokines IL-1β and IL-6; and suppressing the expression of matrix metalloproteinases (MMP)-2 and MMP-9 proteins [17,18]. In addition, SINH is able to inhibit the inflammatory response by reducing oxidative stress [19]. Therefore, SINH has the potential to treat AD.

In view of the problems of SINH with a short half-life, low oral bioavailability, and gastrointestinal irritation caused by high blood concentrations [20,21], this study aimed to explore a new sinomenine hydrochloride delivery system—SINH-liposome-hydrogel (SINH-L-H) system. SINH loaded into liposomes and evenly dispersed in hyaluronic acid colloidal hydrogel take advantage of their excellent biocompatibility and high hydration capacity, enabling the drug to remain on the surface of the skin for a longer period of time [22], improving the bioavailability of the drug and providing benefits for the treatment of atopic dermatitis. Due to their structural properties, the liposomes-in-hydrogel not only act as carriers but also achieve slow drug release and keep the affected area moist, which is crucial for the treatment of dry, inflammatory skin diseases such as AD. In addition, SINH-L incorporated into colloidal hydrogel provided the dual effect of enhanced drug permeability and sustained release to reduce skin lesions and provide antioxidant and anti-inflammatory effects [23]. Therefore, this paper will construct a mouse model of atopic dermatitis, observe and evaluate the therapeutic effect of the SINH-L-H system on atopic dermatitis, explore the mechanism of action of the SINH-L-H system in the treatment of atopic dermatitis, and verify the effectiveness of the SINH-L-H system in the treatment of atopic dermatitis. 

## 2. Results

### 2.1. Molecular Docking of SIN as the Active Structure of SINH

**Figure 2:**
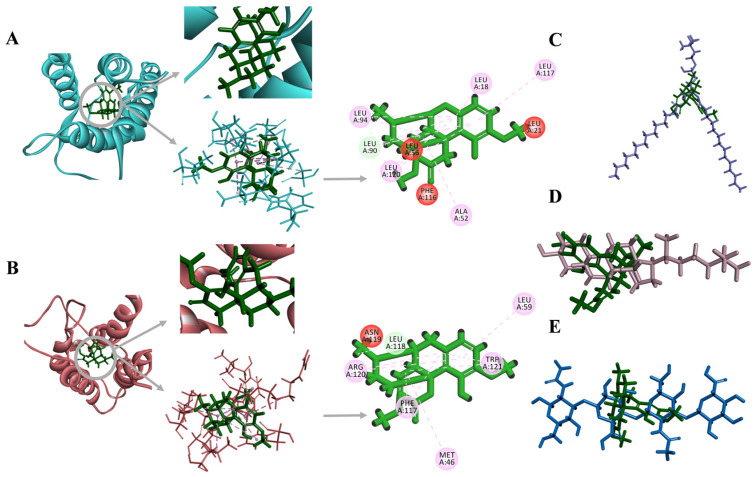
Molecular docking of SIN through Discovery Studio 4.5: (**A**) IL4 and SIN, (**B**) IL2 and SIN, (**C**) Cholesterol and SIN, (**D**) Phospholipid and SIN, (**E**) Hydrogel and SIN.

The computer software Discovery Studio 4.5 was used to predict the interactions between the components of the formula. Through software screening, the three-dimensional structure of proteins IL4 and IL2 combined with SIN was selected, and the results showed that SIN had a good docking effect with IL4 and IL2. There are pi-alkyl and carbon-hydrogen bonds between SIN and IL4 (Figure 2A). There are van der Waals forces, pi-alkyl, and carbon-hydrogen bonds between SIN and IL2, and there is also a pi-pi stacked interaction between molecules (Figure 2B). In the same space, there are carbon-hydrogen bonds and bump interactions between SIN, cholesterol, and phospholipid in different conformations (Figure 2C,D). There is also a bump interaction between SIN and hydrogel (Figure 2E).

### 2.2. Basic Characteristics of SINH-L-H

**Figure 3:**
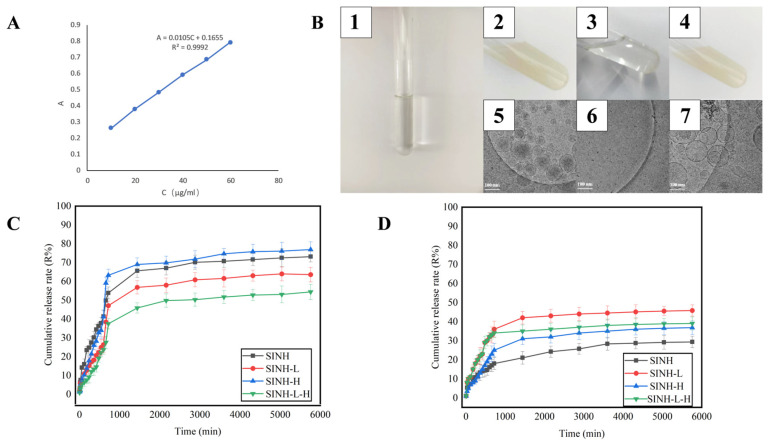
Characteristics of SINH. (**A**) Standard curve of determination of SINH. (**B**) The appearances of SINH, SINH-L, SINH-H, and SINH-L-H (1–4) and the images of SINH-L, SINH-H, and SINH-L-H under the cryo-EM (5–7). (**C**) Cumulative release curve of SINH and its three preparations through the dialysis membrane at different time points. (**D**) Cumulative release curve of SINH and its three preparations through mice skin at different time points.

The absorbance of SINH was measured by an ultraviolet spectrophotometer; SINH at 10–60 μg/mL concentration is proportional to their absorbance (R = 0.9992), and the linear relationship is good (Figure 3A). The SINH-loaded liposomes-in-hydrogel (SINH-L-H) is a creamy white, homogeneous, and fine hydrogel with no viscous lumps and low fluidity, remaining colloidal hydrogel at room temperature (Figure 3B). The liposomes in SINH-L-H were spherical and uniformly dispersed in the hyaluronic acid hydrogel (HA) via Cyro-EM (Figure 3B); the size of the SH-L is about 133.8 ± 14.5. Viscosity measurements showed the viscosity of SINH-L-H was 9.83 ± 0.11 mPa·s at 25 °C, and its pH was 7.27 ± 0.01. The encapsulation rate of SINH-L is 72.27%. 

Stability measurements showed that SH-L-H did not change significantly when stored for 30 days under refrigeration at 4 °C. The appearance and viscosity of SH-L-H did not differ from day 0, indicating that SH-L-H was stable under these conditions. SH-L-H is more variable when stored at ambient conditions: for the first 14 days, the SH-L-H was similar to the initial preparation, creamy white, homogeneous, and fine in texture; on the 21st day, the color of the SH-L-H changed from creamy white to yellowish and the viscosity decreased; on the 30th day, the color of the SH-L-H continued to deepen to yellow, and there was stratification and a decrease in viscosity. The above indicates that SH-L-H is unstable when stored under these conditions and that the drug is easily released from the liposomes and oxidized and discolored (Table 1).

### 2.3. In Vitro/Ex Vivo Dialysis Diffusion Dialysis

The in vitro and ex vivo release profiles of SINH, SINH-L, SINH-H, and SINH-L-H are shown below (Figure 3C,D). It can be seen that the sample from the upper diffusion pool gradually penetrates into the lower diffusion pool as the release time increases. 

In the in vitro diffusion experiment, the release rate of the SH group was greater than the other three groups during the first 11 h. After 11 h, the release rate of SINH in SINH-H began to exceed that of the SINH group, which indicates that the hydrophilic porous structure of SINH-H promoted the release of SINH, so that SINH rapidly passed through the dialysis membrane into the lower diffusion pool, and the release rate of SINH in SINH-H did not show a large change with the increase in time, and the release curve showed that it entered the plateau period at about 12 h, and the final release degree could reach about 77%; the largest amount of SINH released by SINH-L-H occurs at 96 h, up to about 55%, this may be due to the fact that SINH-L-H possesses the three-dimensional mesh structure of HA hydrogels and the phospholipid bilayer of liposomes, and thus SINH is tightly bound in it and cannot be easily released. 

In the ex vivo diffusion experiment, the total diffusion rate was SINH-L > SINH-L-H > SINH-H > SINH, and SINH-L reached the highest permeation efficiency of about 46% at 96 h, while the release rate of SINH only reached 30% at this time, and the liposomes contained phospholipids similar to skin tissues, which were “similarly compatible”. Therefore, the release of SINH in SINH-L is the greatest, while SINH is in the free state and less compatible with the skin, so the amount and speed of penetration through the skin are smaller. SINH-L-H contains phospholipids and HA, which are also contained in skin tissues, and although it can contact a larger area of skin, it also takes time to cross the three-dimensional structure of the phospholipid bilayer and hydrogel, so the diffusion rate of SINH in SINH-L-H is lower than that of SINH-L; the amount and rate of release of SINH in SINH-H with the help of HA is slightly higher than that of SINH. 

Comparing the release profiles of in vitro and ex vivo diffusion, we found that the speed of SINH through the dialysis membrane was faster and the amount of transmission was greater in each group, while the transdermal speed of the drug in each group showed irregularities and significant differences. Dialysis membrane is a homogeneous porous membrane of organic polymers that mainly relies on the osmotic pressure generated by the different concentration gradients on both sides of the membrane to promote the flow of solutes, so the speed and amount of SINH passage mainly depend on the osmotic pressure on both sides of the membrane. The skin includes the dermis and epidermis with abundant cells and blood vessels, and it will take more time for the drug to pass through the skin. The principle of similar compatibility between liposomes and cell membranes means that more SINH in SINH-L and SINH-L-H is deposited in the skin.

### 2.4. Determination of Antioxidant Activity

#### 2.4.1. The Scavenging Rate on Hydroxyl Radicals

**Figure 4:**
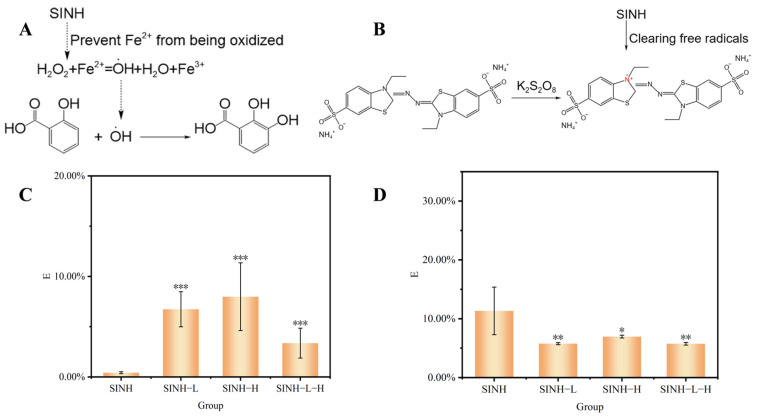
Characteristics and antioxidant activity of SINH and its formulations. (**A**) Fenton reaction mechanism of SINH, (**B**) SINH scavenging ABTS^+^ reaction, (**C**) The effect of SINH, SINH-L, SINH-H, and SINH-L-H on scavenging hydroxyl radicals, (**D**) The effect of SINH, SINH-L, SINH-H, and SINH-L-H on scavenging ABTS radicals (* *p* < 0.05, ** *p* < 0.01, *** *p* < 0.001: Pharmaceutical preparation group vs. SINH group).

In the Fenton reaction, hydroxyl radicals react with salicylic acid to generate 2,3-dihydroxybenzoic acid, which exhibits a special absorption at 510 nm (Figure 4A). SINH had antioxidant activity in the reaction system and could reduce the production of hydroxyl radicals. Therefore, the generation of 2,3-dihydroxybenzoic acid in the reaction system decreased, and the color became lighter. It was indicated that the clearance rate of SINH for 2,3-dihydroxybenzoic acid was directly proportional to the concentration of SINH in the concentration range of 0.2 to 1.0 mg/mL. The IC_50_ of SINH for scavenging hydroxyl radicals was about 0.7034 mg/mL.

In the antioxidant reaction experiment, the drugs in SINH-L-H were surrounded by liposomes and hydrogels, and SINH was difficult to release. However, because of the synergistic effect of the antioxidant capacity of liposomal hydrogel and SINH itself, the clearance rate of SINH-L-H was higher than that of SINH. At the concentration of 0.2 mg/mL, the scavenging rates of SINH, SINH-L, SINH-H, and SINH-L-H on hydroxyl radicals were about 0.42 ± 0.1%, 6.73 ± 1.7%, 7.98 ± 3.4%, and 3.36 ± 1.5%, respectively. The clearance rate of SINH was lower than that of the same concentration formulation (Figure 4B). In the Fenton reaction, the removal effect of SINH-L-H was lower than that of SINH-L and SINH-H but higher than that of SINH. This showed that SINH-L-H had better stability and durability while maintaining the sinomenine’s effect so that the drug could work in the body for a longer time.

#### 2.4.2. The Inhibitory Rate on ABTS Radicals

The 1:1 mixture reaction of ABTS and K_2_S_2_O_8_ produced stable cationic free radicals ABTS^+^. The maximum absorption wavelength of ABTS^+^ is 734 nm, and the reaction between antioxidants and ABTS^+^ will cause the solution to fade (Figure 4C). The stronger the antioxidant capacity, the lighter the color of the solution, and the higher the free radical scavenging rate.

The scavenging ability of SINH on ABTS^+^ was directly proportional to the concentration between 0.001 and 0.02 mg/mL, and the higher the concentration, the stronger the scavenging ability. The IC_50_ value of SINH was about 0.0115 mg/mL. The reaction steps of SINH, SINH-L, SINH-H, and SINH-L-H with the same concentration of 0.001 mg/mL have clearance rates of 11.33 ± 4.21%, 5.76 ± 0.32%, 6.96 ± 0.41%, and 5.74 ± 0.36% for ABTS^+^, respectively (Figure 4D). The clearance rate of ABTS^+^ in the preparations was lower than that of SINH. The clearance of ABTS^+^ was lower than that of SINH. This was because SINH was encapsulated in the liposomal hydrogel, and the release rate of SINH was slow. For a certain period of time, the free radical scavenging ability of SINH-L-H was weaker than that of SINH. But SINH-L-H had a slow release effect, which made it have a long-term effect. Therefore, SINH-L-H had the ability for long-term, slow release.

### 2.5. The Therapeutic Effects on AD Mice

#### 2.5.1. Measurements Results of Skin Irritation of SINH-L-H

In the single-dose skin irritation test, no mice in the B-L-H and SINH-L-H groups showed any skin changes within 72 h after administration (Table 2). In multiple skin irritation experiments, the mice in the B-L-H (Blank-loaded liposomes-in-hydrogel) and SINH-L-H groups did not show any erythema or edema within 7 days after administration, indicating that the formula matrix we selected was safe and non-toxic (Table 3). In terms of scoring results, B-L-H and SINH-L-H were safe because of no irritation to intact mouse skin.

#### 2.5.2. General Condition of Mice and Manifestation of Skin Lesions

**Figure 5:**
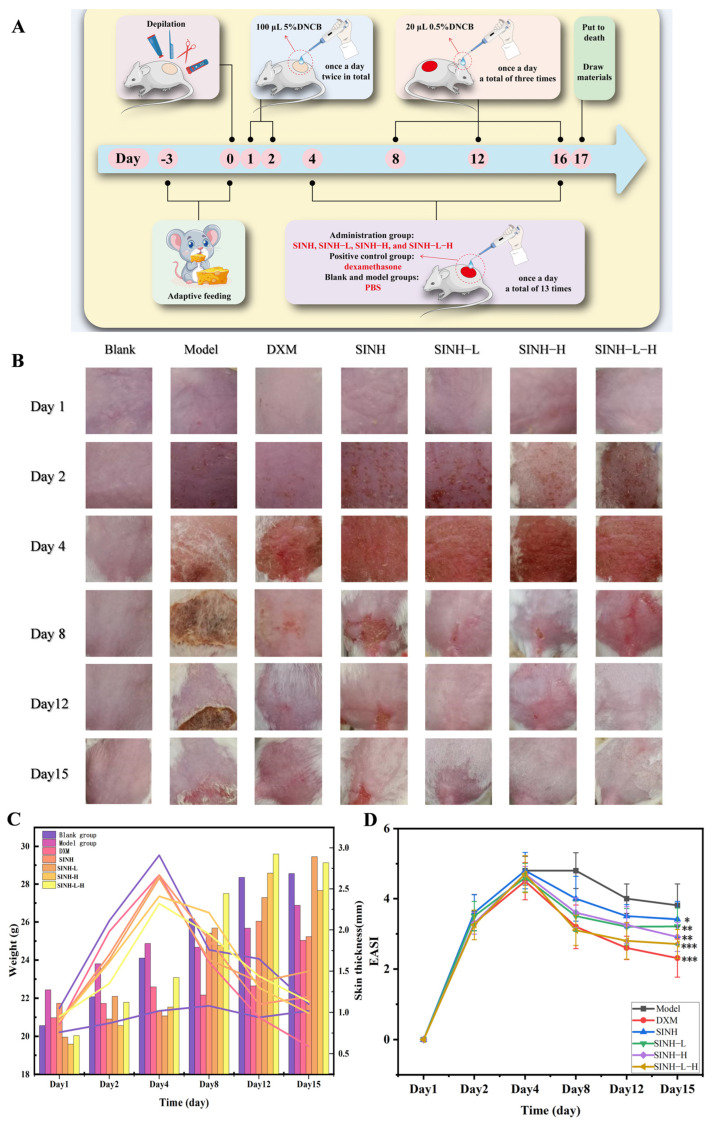
Pharmacological activity of SINH, SINH-L, SINH-H, and SINH-L-H. (**A**)The establishment of an eczema mouse model and the time point of drug administration, (**B**) Skin lesions in mice, (**C**) Changes in body weight and skin thickness of mice (*n* = 10), (**D**) Degree of skin lesion score (compared with the model group: * *p* < 0.05, ** *p* < 0.01, *** *p* < 0.001).

The establishment of the AD mouse model and the time point of drug administration according to Figure 5A. The body weight and skin thickness of each group of mice are shown in Figure 5B, and the skin healing is shown in Figure 5C. The mice in the blank group showed an increasing trend in body weight and skin thickness, with smooth hair, good condition, and no abnormal behavior; the ears were red and transparent, with an intact appearance and clearly visible blood vessels. The body weight of mice in the other groups peaked on the fourth day and decreased thereafter, among which the body weight of mice in the positive control group with ointment decreased more rapidly, presumably due to the side effects caused by dexamethasone as a kind of glucocorticoids. After 2,4-dinitrochlorobenzene (DNCB) excitation twice, erythema appeared on the dorsal skin of mice in each group and gradually thickened and dried, with scabs appearing, and mice scratched frequently and became agitated. On the 4th day, the erythema and thickening of the skin of the mice in each group were gradually reduced, and the skin of the mice in the positive control group was obviously thinner, with slight folds and erythema and no hair growth at the excitation site. As shown in Figure 5D, compared with the model group, the skin injury scores of mice in the model group were significantly higher than those in the normal control group, and the skin scores of mice in the positive control group were significantly lower than those in the SINH-L-H group.

#### 2.5.3. Ear Swelling in Mice

**Figure 6:**
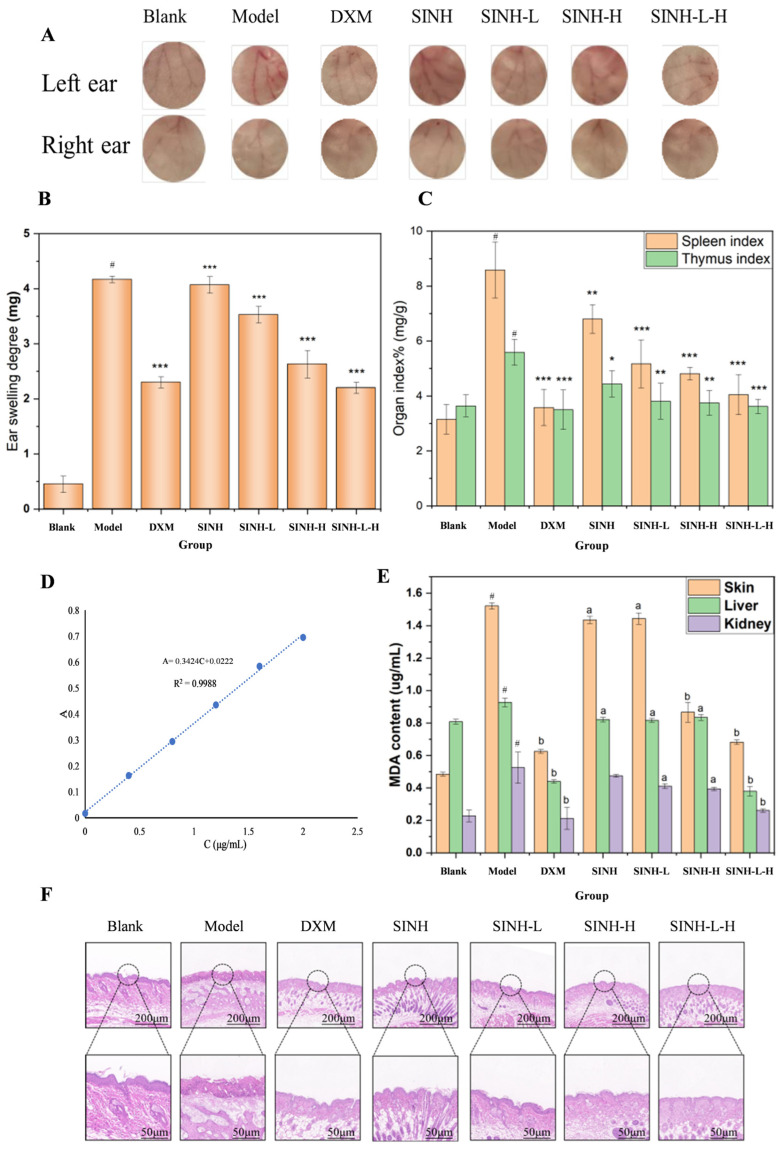
Effect of SINH-L-H on eczema mice. (**A**) Effect of SINH-L-H on mice ears in eczema mice; (**B**) Effect of SINH-L-H on ear swelling in AD mice (Compared with the blank group: # *p* < 0.001; compared with the model group: *** *p* < 0.001); (**C**) Effect of SINH-L-H on the organ index of AD mice (# *p* < 0.001: model group vs. blank group; * *p* < 0.05, ** *p* < 0.01, *** *p* < 0.001: drug group vs. model group); (**D**) MDA standard curve; (**E**) Effect of DNCB induction on MDA content in various tissues of mice ($\bar{X}\pm SD$, *n* = 10) Note: Compared with blank group: # *p* < 0.001; compared with model group: a *p* < 0.01, b *p* < 0.001; (**F**) Pathological skin changes on the back of each group of mice.

Except for the blank group, the ears of mice in the other groups showed swelling and crusting after excitation, and the symptoms significantly improved after SINH-L-H administration. Compared with the blank group, the swelling and crusting status of the remaining groups increased to varying degrees, with the most significant change in mice’s status in the model group. Compared with the model group, the mice were administered to the SINH-L-H group with the most significant ear changes (Figure 6A,B).

#### 2.5.4. Mouse Organ Index

The effect of SINH-L-H on AD was initially evaluated by organ indices, including the spleen index and thymus index, and the results are shown in Figure 6C. Compared with the blank group, the spleen index and thymus index of mice in the model group increased, and the differences were statistically significant; compared with the model group, the spleen index and thymus index of mice using SINH, SINH-L, SINH-H, and SINH-L-H decreased, and the effect of using SINH-L-H was almost similar to that of using dexamethasone, indicating that SINH and its three preparations all have good efficacy on AD.

#### 2.5.5. Malondialdehyde (MDA) in Mouse Organ

MDA, one of the products of lipid peroxidation, is one of the important indexes to measure the degree of oxidative damage. The content of MDA in each mouse tissue is shown in Figure 6D,E. Compared with the blank group, the content of MDA in the skin and liver of mice in the model group increased with significant differences, and the content of MDA in the kidney also increased. All MDA levels in the skin, liver, and kidney of mice in the SINH, SINH-L, SINH-H, and SINH-L-H groups also decreased, with the most pronounced decrease in mice in the SINH-L-H group, indicating that SH-L-H was the most effective in AD among these three groups.

#### 2.5.6. HE Staining

The results of HE staining of the dorsal skin of mice in each group are shown in Figure 6F. Compared with the blank group, the dorsal skin of the model mice showed obvious hyperplasia, significant thickening of the epidermal layer, intracellular and intercellular edema, and a large amount of inflammatory infiltration around the blood vessels in the dermis, showing a severe inflammatory state overall. In the control group, the structure of each layer was more complete with clear borders; the epidermis was not obviously keratinized; the spiny layer was mildly hyperplastic; there was mild intracellular and intercellular edema; and the inflammatory infiltrate around the blood vessels in the dermis was mild. The rest of the administered groups were less symptomatic than the model group, showing varying degrees of epidermal layer thickening, intracellular and intercellular edema, and vascular inflammatory infiltration, with the SINH-L-H group producing the mildest manifestations (Figure 6).

## 3. Discussion

AD is a common skin disease that can have a serious impact on a patient’s life. It is currently considered to have a strong correlation with susceptibility genes, epidermal barrier dysfunction, and immune dysfunction. The AD model was established by DNCB, leading to epidermal barrier dysfunction, and the therapeutic effect of SINH-L-H on AD was studied.

Liposomes were reported to be an excellent drug carrier, which could load drugs for the treatment of skin injuries, and hydrogels could clear wound exudates, provide a moist environment, and stimulate and guide tissue regeneration, which could be used as external materials for wounds [24,25]. In this study, hyaluronic acid was selected for the colloidal hydrogel matrix. Hyaluronic acid itself is a polysaccharide and has additional characteristics that make it very suitable for drug delivery systems, such as biocompatibility, antiviral, antibacterial, and anti-tumor properties. The combination of liposomes and hyaluronic acid to load drugs could form an excellent delivery system to enable the drug to be delivered to its target. The successful preparation of SINH-L-H had the characteristics of a slow and controlled release. In addition, SINH-L-H prepared in the study had stable properties, good dispersion, and an excellent dermal penetration effect, which was in line with the expected effect.

The onset of AD could be caused by multiple factors. Abnormal Notch signaling pathways can induce the development of severe AD, while normal Notch signaling pathways can inhibit the production of thymic stromal lymphopoietin (TSLP) in keratinocytes and alleviate the pathogenesis of AD [26]. Pro-inflammatory cytokines can promote the occurrence of AD, including TSLP, IL-25, and IL-33 [27]. Th2 cells are derived from the immune differentiation of T cells and serve as pro-inflammatory mediators in AD. Activate Th2 cells, promote specific cytokine-related inflammation (IL-4, IL-5, IL-13), an increase in eosinophils, and the production of immunoglobulin E (IgE) [28]. B cells participate in T cell activation and Th2 production and differentiate into cytokine-producing cells that may alter the differentiation of effector T cells [29]. One of the main signs of AD is an increase in serum total IgE levels. Activated Th2 cells release IL-4 and IL-13, which promote B cells to produce IgE antibodies through the signal transducer and transcriptional activator (STAT) pathway [30]. The various pathogenic factors mentioned above can serve as targets for the treatment of AD. 

**Figure 7:**
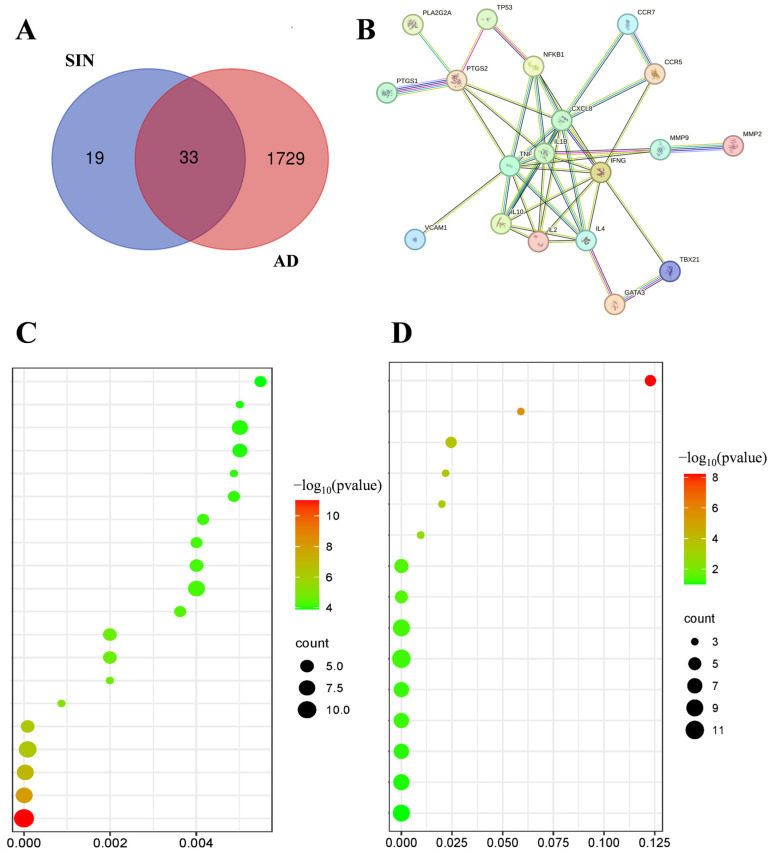
Molecular formation and target screening of drug systems: (**A**) Venn diagram of AD and SIN targets, (**B**) Bubble diagram of target PPI, (**C**,**D**) GO and KEGG enrichment.

In order to better predict the target of action between AD and SIN, we used Genecards (GeneCards—Human Genes|Gene Database|Gene Search (https://www.genecards.org/), retrieved from 15 January 2023) to screen out the target of disease and drug action and created a Venn diagram to find the intersection of the target of action (Figure 7A). DIVID (DAVID Functional Annotation Bioinformatics Microarray Analysis (ncifcrf.gov), retrieved from 16 January 2023) was used to find the relevant pathways of the intersection genes, and bioinformatics (bioinformatics.com.cn) was used to enrich the pathways and find the best pathway (Figure 7C,D). At the same time, use STRING (STRING: functional protein association networks (string-db.org), retrieved from 17 January 2023) to create a Protein-Protein interaction network (PPI) graph of the intersecting genes and identify the genes that have the most significant impact (Figure 7B). Finally, the most relevant gene targets were extracted from the screened pathways and rescreened with significant genes in STRING. IL2 and IL4 were selected as the predicted targets for SIN treatment of AD.

Studies have shown that there is an excess of reactive oxygen species in the skin of atopic dermatitis patients, and these reactive oxygen species can cause high oxidative stress, resulting in oxidative damage to skin cell DNA and proteins, further aggravating skin inflammation and damage [31,32]. SINH, SINH-L, SINH-H, and SINH-L-H are able to remove reactive oxygen species, which helps reduce oxidative damage to cells due to the accumulation of reactive oxygen species. By reducing the accumulation of reactive oxygen species, SINH-L-H may help reduce oxidative damage to skin cells and improve skin barrier function, thereby relieving the symptoms of atopic dermatitis. Therefore, SINH-L-H is a potential drug for the treatment of AD.

## 4. Materials and Methods

### 4.1. Materials

Sinomenine hydrochloric (≥98%, China), cholesterol (AR, Sinopharm Chemical Reagent Co., Ltd., Shanghai, China), soy lecithin (Shanghai Jinsong Industry Co., Ltd., Shanghai, China), absolute ethanol (AR, Shanghai RichJoint Chemical Reagents Co., Ltd., Shanghai, China), hyaluronic acid (HA, 99%, Shandong Xiya Reagents Co., Ltd., Linyi, China), 1,1-diphenyl-2-picrylhydrazyl (DPPH, 98%, Shanghai Yuanye Biology Science and Technology Co., Ltd., Shanghai, China), H_2_O_2_ (AR, Shanghai SuYi Chemical Reagent Co., Ltd., Shanghai, China), FeSO_4_·7H_2_O (AR, Sinopharm Chemical Reagent Co., Ltd., Shanghai, China), thiobarbituric acid (TBA, 98%, Shanghai Yuanye Biology Science and Technology Co., Ltd., Shanghai, China), trichloroacetic acid (TCA, AR, Tianjin Damao Chemical Reagent Factory, Tianjin, China), 2,4-dinitrochlorobenzene (DNCB, Shandong Xiya Reagents Co., Ltd., Linyi, China), 2,2′-Azinobis-(3-ethylbenzthiazoline-6-sulphonate) (ABTS, 98%, Shanghai Macklin Biochemical Technology Co., Ltd., Shanghai, China), K_2_S_2_O_8_ (99.5%, Macklin Biochemical Technology Co., Ltd., Shanghai, China), phosphate buffered saline (PBS).

### 4.2. Animals

Healthy Kunming mice (female, 20 ± 2 g) were provided by the Animal Experimental Center of the Anhui University of Traditional Chinese Medicine (Hefei, China). All animal experiments conform to the guidelines approved by the Ethics Committee of the Anhui University of Traditional Chinese Medicine (Hefei, China). Animals are kept under constant environmental conditions (25 ± 2 °C, 40–70% relative humidity), and provided with unlimited supplies of food and water.

### 4.3. Preparation SINH-L, SINH-H, and SINH-L-H

**Figure 8:**
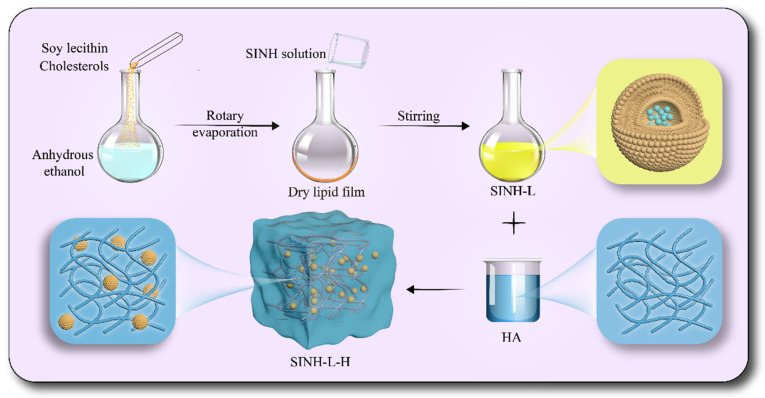
Preparation process of SINH-L-H.

The synthesis of SINH-L, SINH-H, and SINH-L-H was adopted from our previous work as cited [23] (Figure 8). Took 0.3 g of phospholipids and 0.1 g of cholesterol in a rotary evaporation flask, added the appropriate amount of absolute ethanol to dissolve, fixed the flask on a rotary evaporator to rotated and evaporated to form a thin film, set the temperature at 70 °C, vacuum pressure at 70 kPa, speed at 100 r/min until the absolute ethanol evaporated completely. Then, 10 mL of 2 mg/mL of SINH solution was added to the film and rotated and hydrated for 30 min to form a milky white suspension. The suspension was sonicated for 10 min and filtered across the 0.22 μm microporous membrane three times to obtain SINH-L. 0.1 g HA was added to the prepared 10 mL SINH-L suspension and stirred at the speed of 100 rpm for 12 h with a magnetic stirrer till HA naturally swelled to form a uniform hydrogel (SINH-L-H). 0.1 g of HA was also added to 10 mL of SINH solution, and the same was used to form SINH-H. In addition, blank liposomes (B-L) with PBS instead of SINH solution, blank liposome hydrogels (B-L-H) with B-L instead of SINH-L, and blank HA hydrogels (B-H) with PBS instead of SINH solution were prepared.

### 4.4. Quality Evaluation of SINH-L

#### 4.4.1. Appearance and Morphology of Cryo-EM

The prepared SINH-L suspension was placed in a transparent glass test tube to observe its appearance. An appropriate amount of prepared SINH-L was mixed with a turbine apparatus, and the morphological characteristics of SINH-L were observed with cryo-EM.

#### 4.4.2. Particle Size and Zeta Potential

First, we applied the Malvern potential particle simeter (*n* = 3) to measure the particle size of SINH-L and the electrophoretic mobility of SINH-L in electric fields. The electrophoretic mobility was then converted to the Zeta potential value according to the Smoluchowski equation.

#### 4.4.3. Encapsulation Rate and Drug Load

In this experiment, the drug amount of free sinomenine hydrochloride in SH-L suspension was obtained by the high-speed centrifugal method, and the drug amount of total sinomenine hydrochloride in SINH-L suspension was obtained by the ethanol demulsification method: 1 mL SINH-L suspension was precisely measured and diluted by adding an appropriate amount of PBS solution. After centrifugation at 15,000 RPM for 10 min, the supernatant was taken to measure the dosage of free SINH (*W*_1_) at 262 nm. In addition, 1 mL SINH-L suspension was precisely measured and placed in a 10 mL volumetric bottle; anhydrous ethanol was added to the scale line, and the volumetric bottle was placed in a CNC ultrasonic cleaner for 30 min for ultrasonic demulsification. An appropriate amount of demulsified solution was taken and measured at 262 nm to measure the dosage of total SINH (*W*_2_). Calculate the *EE* of SINH-L according to the following Equation:

$$EE=\frac{\left(W_{2}-W_{1}\right)\times 100\%}{W_{2}}$$

Among them, *W*_1_ represents the amount of free SINH in liposome suspension, and *W*_2_ represents the amount of total SINH in liposome suspension.

### 4.5. Molecular Docking

The 2D molecular structures of SIN, hyaluronic acid hydrogel, phospholipid, and cholesterol were obtained from PubChem. They were imported into Discovery Studio 4.5 and exported as small-molecule compound files in SDF format. Then, we downloaded the core target proteins IL4 and IL6 from the UniProt database and selected the appropriate protein structure. Finally, after ligand extraction, hydrogenation, and water extraction, the docking program of Discovery Studio 4.5 was used for docking between small molecules and therapeutic targets. A molecular docking pattern diagram was achieved.

### 4.6. Ex Vivo/Ex Vivo Dialysis Diffusion Dialysis of SH-L-H

We performed this experiment using the Franz diffusion cell. For in vitro diffusion: first fill each diffusion cell with diffusion solution (PBS) and add a stirrer of appropriate size. A dialysis membrane of appropriate size was fixed in the middle of the upper and lower cells of the diffusion cell, and 1 mL of SH, SH-L, SH-L-H, and their corresponding blanks were added to the upper cell, respectively. At 5 min, 30 min, 60 min, 120 min, … 720 min, 1440 min, 2160 min, and 2880 min, 2 mL of samples were aspirated and immediately supplemented with the same volume of PBS solution. The absorbance was measured at 262 nm using a UV spectrophotometer and the corresponding cumulative drug release rates were calculated according to the Equation.

For the ex vivo skin dialysis experiments, we used the dorsal skin of Kunming mice, and the other operations were the same as for dialysis membrane diffusion.

$$R\left(\%\right)=\frac{C_{n}\times V_{n}+\sum_{i=1}^{n-1}C_{i}\times V}{Q_{t}}\times 100\%$$

where “*R*” is the cumulative drug release rate of SH at the different sampling points, “*C_n_*” is the mass concentration of SH at the nth time point (μg/mL), “*V_n_*” is the volume of the solution sampled at the nth time point (mL), “*C_i_*” is the mass concentration of SH in the receiving solution at the sampling point (*i* ≤ *n* − 1) (μg/mL), “*V*” is the total volume of the lower cells (mL), and “*Q_t_*” is the theoretical drug content (2000 μg/mL).

### 4.7. Determination of Antioxidant Activity of SINH-L-H

#### 4.7.1. The Scavenging Rate on Hydroxyl Radicals

SINH (0.2, 0.4, 0.6, 0.8, and 1.0 mg/mL) was added to the Fenton reaction system with salicylic acid. The experimental operation was as follows: in the sample group, add 1 mL FeSO_4_ solution, 1 mL salicylic acid solution, and 1 mL H_2_O_2_ solution into 1 mL SINH successively to measure the absorbance (*A*_1_); in the control group, add 1 mL of salicylic acid and 2 mL water to 1 mL SINH to measure the absorbance (*A*_2_); in the blank group, add 1 mL FeSO_4_ solution, 1 mL salicylic acid solution and 1 mL H_2_O_2_ solution into 1 mL water to measure the absorbance (*A*_3_). The solution of each group was mixed evenly and reacted in a 37 °C water bath for 30 min. The absorbance at a wavelength of 510 nm was measured. The same operation was used for SINH-L-H, SINH-H, and SINH-L of 0.2 mg/mL as SINH mentioned above.

$$E\%=\frac{A_{3}-\left(A_{1}-A_{2}\right)}{A_{3}}\times 100\%$$

Calculate the clearance rate and IC50 of SINH and its preparations on hydroxyl radicals based on changes in the absorbance.

#### 4.7.2. The Inhibitory Rate on ABTS Radicals

Mix the ABTS aqueous solution and K_2_S_2_O_8_ solution (1:1) evenly, and react in a dark place for 12 h. Dilute the ABTS mixture 30 times so that the absorbance of the solution at 734 nm is within the range of 0.7–0.8. Take 0.5 mL of SINH with different concentrations (0.001, 0.005, 0.01, 0.02, 0.03 mg/mL) and place it in a reaction vessel. Add 2 mL of the 30 times diluted ABTS mixture as the sample group (*A_S_*) and react in the dark at room temperature for 5 min. Then measure the absorbance at 734 nm. The control group (*A_C_*) added 2 mL of PBS to SINH, while the blank group (*A*_0_) added 2 mL of ABTS mixture to 0.5 mL of PBS, and then measured the absorbance using the same procedure.

$$E\%=\frac{A_{0}-\left(A_{S}-A_{C}\right)}{A_{0}}\times 100\%$$

SINH-L, SINH-H, and SINH-L-H with a concentration of 0.001 mg/mL, replace the SINH position with formulations and perform the same operation. Finally, based on the measurement results, calculate IC_50_ and the clearance rate of the samples.

### 4.8. The Therapeutic Effect of SINH-L-H on AD 

#### 4.8.1. Skin Irritation of SINH-L-H

Forty healthy Kunming mice (20 ± 2 g) were provided by the Experimental Animal Center of Anhui University of Chinese Medicine under license No. SYXK (SU): 2020-0009. This research protocol has been approved by the Animal Care and Use Committee of Anhui University of Traditional Chinese Medicine (AHUCM mouse-2023136). The mice were randomly divided into a single-dosing test group and a multiple-dosing test group.

The back hair of the mice was removed before the experiment began. The mice were divided into three groups, one group was evenly applied with 0.3 mL of B-L-H, one group was evenly applied with 0.3 mL of SINH-L-H, and the other group was not treated as a control group. After application, cover the affected area with gauze and secure it with medical pressure-sensitive tape. The erythema and edema, pigmentation, bleeding, and skin roughness were observed at 1 h, 24 h, 48 h, and 72 h after removal of the gauze. 

The skin irritation test after multiple doses was administered in a similar manner to the single dose, with the exception that it required 1 week of continuous dosing at the same site for the same duration and amount of dose each time. The gauze and medical pressure-sensitive tape were also removed 6 h after administration and the site was washed with gauze soaked in warm water. Observe the skin condition of the mice 1 h after each removal of the gauze. Score according to Table 4 and Table 5, (at the same time do the individual project score and then do the comprehensive score).

#### 4.8.2. Establishment of AD Model and Administration

The healthy Kunming mice were domesticated in a specific pathogen-free environment for 3 days. One day before the experiment, shave skin hair with an area of about 2 cm × 2 cm on the back. The shaved back skin was treated with 100 μL 5% DNCB solution (a mixture of acetone:olive oil = 3:1 as solvent) on the first and second days, and the blank group was replaced with the same volume of acetone/olive oil mixture after sensitization. After the first induction, the mice were fed normally with an interval of 5 days. The second sensitization, the skin of the ear was applied with a 0.5% DNCB solution every 3 days, for a total of 3 times. The treatment group was treated with SINH, SINH-L, SINH-H, and SINH-L-H, respectively, starting on the 4th day (if sensitized on the same day, dosed 2 h before sensitization) for 12 days, and the mice of the blank and model groups were treated with equal amounts of PBS, while DXM group was treated with dexamethasone (DXM) as a positive control. The mice were fasted for 12 h after the final administration. The ear pieces, spleen, serum, and dorsal skin of the mouse were collected for subsequent tests after execution (Figure 4A). 

#### 4.8.3. Degree of Skin Lesion Score (EASI Score) in AD Mice

During the modeling process, the mice were observed for changes in their physiological status, including activity, body weight, changes in skin thickness, fur color, diet, and water intake. Skin thickness is measured using a sebum thickness meter (also known as a skin fold caliper) to measure the thickness of the skin and underlying adipose tissue by picking up skin folds. The mice were scored 12 h after the last administration according to the Eczema Severity Index (EASI) scale. The scoring criteria included erythema, papules, desquamation, crusting, and exudate, each of which was divided into three degrees of mild, moderate, and severe and expressed in different data. The scoring criteria were as follows, and the total score was the mouse skin lesion score (Table 6). Based on this scale, the establishment of the AD model and the intervention of SINH, SINH-L, SINH-H, and SINH-L-H on this model were obtained. 

#### 4.8.4. Determination of Ear Swelling Rate in Mice

After the mice were executed, the right and left ear pieces (about 8 mm) were immediately removed with a punch and weighed on an analytical balance, and the ear swelling rate of the mice was calculated according to the following formula.

$$\mathrm{Ear}\;\mathrm{swelling}\;\mathrm{rate}\%=\frac{\mathrm{Right}\;\mathrm{ear}\;\mathrm{mass}-\mathrm{Left}\;\mathrm{ear}\;\mathrm{mass}}{\mathrm{Left}\;\mathrm{ear}\;\mathrm{mass}}\times 100\%$$

#### 4.8.5. Measurement of Organ Index in Mice

After the mice were executed, the thymus and spleen tissues were separated and quickly weighed, and the thymus (spleen) index was calculated according to the formula as follows:

$$\mathrm{Spleen}\;\left(\mathrm{thymus}\right)\;\mathrm{index}=\frac{\mathrm{Spleen}\;\left(\mathrm{thymus}\right)\;\mathrm{mass}\;\left(\mathrm{mg}\right)}{\mathrm{Body}\;\mathrm{mass}\;\left(\mathrm{mg}\right)}$$

#### 4.8.6. Determination of MDA Content in Organ

Mouse liver and kidney were taken to prepare 10% organ homogenate. The content of MDA in mouse organs was determined according to the following steps: (1) The experiment was divided into the blank, model, DXM, SINH, SINH-L, SINH-H, and SINH-L-H groups. (2) 1 mL of skin, liver, and kidney homogenate from each group was added to 3 mL of TBA working solution and placed in a water bath at 95 °C for 40 min. (3) After removal and cooling to room temperature with running water to stop the reaction, the supernatant was centrifuged at 4000 r/min for 8 min and the absorbance value was measured at 532 nm. (4) The absorbance values obtained were substituted into the MDA content standard curve to calculate the MDA content in the tissue and determine the degree of oxidation of the tissue. The MDA content in the skin, liver, and kidney were determined the same as above.

#### 4.8.7. Hematoxylin and Eosin Staining

After removing the skin tissue from the back of the mice, the normal saline was rinsed and quickly fixed in 4% formalin buffer and embedded in paraffin 24 h later. Skin sections with a thickness of 5 μm made by the section mechanism and stained with hematoxylin-eosin (HE) were observed in the skin histopathological characteristics of mice. 

#### 4.8.8. Statistical Methods

All data were expressed as mean ± standard deviation (x ± s), one-way ANOVA was used, and the LSD method was used for two-way comparison between groups, and the test level was α = 0.05, and the difference was considered statistically significant at *p* < 0.05. 

## 5. Conclusions

To sum up, SINH was encapsulated in liposomes and then evenly dispersed into the HA hydrogel matrix to form SINH-L-H, which could increase the residence time on the skin surface to improve the bioavailability of SINH to treat AD. The experimental results showed that SINH-L-H had uniform texture, good stability, and a slow release effect. SINH-L-H was a safe preparation based on its non-irritating effect on the skin. In the antioxidant test, SINH-L-H showed strong scavenging ability on hydroxyl free radicals and ABTS free radicals, and the scavenging ability of SINH was positively correlated with the concentration of SINH, indicating that SINH had a good antioxidant effect. For AD in mouse models, the use of SINH-L-H significantly alleviated the symptoms of AD and reduced the amount of MDA in the skin. SINH penetrated into the skin, depending on the liposomes-in-gel delivery. SINH-L-H has a good transdermal effect. SINH-L-H was a safe and effective new dosage form for the treatment of AD and provided a novel carrier for the clinical application of SINH.

## Figures and Tables

**Table 1:** Determination results of SH-L-H 4 °C cold storage experiment and normal temperature storage experiment.

Storage Temperature	Storage Period (d)	Characters	Exterior	Viscosity (_mPa·s)
4 °C	0	Milky white, even and delicate	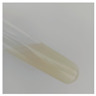	9.84 ± 0.14
	7	Milky white, even and delicate	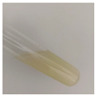	9.76 ± 0.08
	14	Milky white, even and delicate	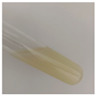	9.80 ± 0.03
	21	Milky white, even and delicate	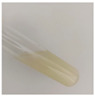	9.79 ± 0.05
	30	Milky white, even and delicate	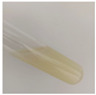	9.82 ± 0.03
Normal temperature	0	Milky white, even and delicate	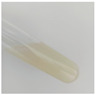	9.84 ± 0.02
	7	Milky white, even and delicate	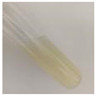	9.73 ± 0.01
	14	Milky white, even and delicate	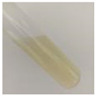	9.70 ± 0.03
	21	Pale yellow, even and delicate	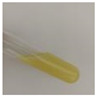	9.65 ± 0.04
	30	Yellow, layered	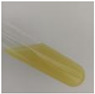	9.57 ± 0.06

**Table 2:** Skin irritation score of a single administration.

Time	Group				Irritation Intensity
	B-L-H Skin Status	B-L-H Skin Score	SINH-L-H Skin Status	SINH-L-H Skin Score	
1 h	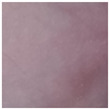	0	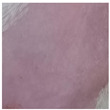	0	None
24 h	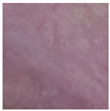	0	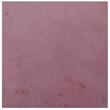	0.2	None
48 h	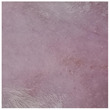	0	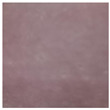	0	None
72 h	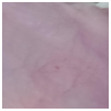	0	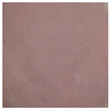	0	None

**Table 3:** Skin irritation score of multiple administration.

Time	Group				Irritation Intensity
	B-L-H Skin Status	B-L-H Skin Score	SINH-L-H Skin Status	SINH-L-H Skin Score	
1 d	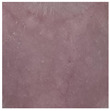	0	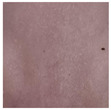	0	None
2 d	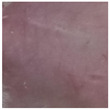	0	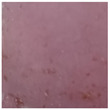	0.2	None
3 d	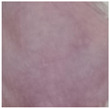	0	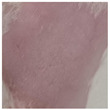	0	None
4 d	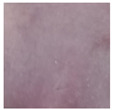	0	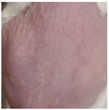	0	None
5 d	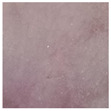	0	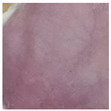	0	None
6 d	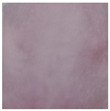	0	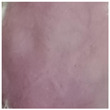	0	None
7 d	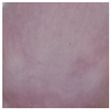	0	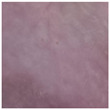	0	None

**Table 4:** Skin irritation response score.

Skin Reaction	Degree of Response	Score
Erythema and eschar	Erythema free	0
	Slight erythema (barely visible)	1
	Moderate erythema	2
	Erythema severa	3
	Purplish red erythema to slight eschar formation	4
Edema condition	No edema	0
	Slight edema (barely visible)	1
	Mild edema (edema bulge clearly defined)	2
	Moderate edema (edema bulge about 1 mm)	3
	Severe edema (swelling of more than 1 mm, extended range)	4
Highest score		8

**Table 5:** Evaluation criteria of skin irritation intensity.

Average Score	Irritation Intensity
0–0.49	Non-irritant
0.5–2.99	Mild irritation
3–5.99	Moderate irritation
6–8	Strong irritation

**Table 6:** Score of eczema skin lesion in mice (EASI Score).

Skin Condition	Degree of Response	Points
No obvious skin injury was observed by naked eye	None	0
Visual inspection requires careful examination to see the lesions	Mild	1
The lesions are clearly visible to the naked eye	Medium	2
The lesions were severe and very visible	Severe	3
The score between the various symptoms can be recorded as a half grade (0.5)		

## Data Availability

Data will be made available on request.
